# The complete chloroplast genome of *Eurya loquaiana* (Pentaphylacaceae)

**DOI:** 10.1080/23802359.2020.1814174

**Published:** 2020-09-03

**Authors:** Qian Wang, Yan-Yan Ao, Shu-Dong Zhang, Bo Ding, Hong-Ping Deng

**Affiliations:** a Chongqing Key Laboratory of Plant Resource Conservation and Germplasm Innovation, Institute of Resources Botany, School of Life Sciences, Southwest University, Chongqing, China; b School of Biological Science and Technology, Liupanshui Normal University, Liupanshui, Guizhou, China; c Biotechnology Research Center, Southwest University, Chongqing, China; d Chongqing Research Center for Low Carbon and Ecological Environment, Chongqing Academy of Science & Technology, Chongqing, China

**Keywords:** Chloroplast genome, *Eurya loquaiana*, phylogenetic analysis, Pentaphylacaceae

## Abstract

The complete chloroplast (cp) genome of *Eurya loquaiana* Dunn. has been reported in this study. The cp genome has a total length of 157,218 bp with the typical quadripartite structure, containing two inverted repeats (IRs) of 25,883 bp separated by a large single-copy (LSC) region of 87,248 bp and a small single-copy (SSC) region of 18,204 bp. The whole cp genome of *E. loquaiana* contains 128 genes, including 83 protein-coding genes, 37 tRNAs genes, and 8 rRNAs. The phylogenetic result showed that *E. loquaiana* is sister to *E. alate*.

*Eurya* Thunberg (Pentaphylacaceae) contains about 130 species worldwide, and mainly distributed in subtropical, tropical Asia and pacific islands (Min and Bartholomew 2007). The plants of this genus are usually evergreen shrubs with small fragrant flowers (Min and Bartholomew 2007). They are important nectariferous plants with certain ornamental and medicinal values (Chen 2010; Song et al 2017). In this study, the chloroplast (cp) genome of *E. loquaiana* Dunn. was first reported. To confirm the relationship within Pentaphylacaceae, the phylogenetic tree was reconstructed with the complete cp genome sequences, which will provide useful information for the further study of this genus.

The fresh and clean leaves of *E. loquaiana* were collected from Beibei district of Chongqing, China (N29°49′49.36″, E106°22′46.58″, 856 m). The voucher specimen (yanyanao20191001) was deposited in the herbarium of Southwest University (SWCTU). The total genomic DNA was extracted and used for sequencing on Illumina HiSeq 4000 platform at the Beijing Novogene Bioinformatics Technology Co., Ltd. (Nanjing, China). To obtain the complete cp genome, SPAdes (Bankevich et al. 2012) was used to *de novo* reassemble the raw data. The complete genome sequence was annotated using PGA (Qu et al. 2019) with manual adjustments. The sequence of cp genome was deposited in the GenBank (accession numbers MT038209).

The cp genome of *E. loquaiana* is 1,57,218 bp in size with the typical quadripartite structure of angiosperms, containing a couple of inverted repeats (IRs) of 25,883 bp separated by a large single-copy (LSC) region of 87,248 bp and a small single-copy (SSC) region of 18,204 bp. The total GC content of the whole sequence is 37.3%. The cp genome of *E. loquaiana* contains 128 genes, including 83 protein-coding genes (PCGs), 37 transfer RNA (tRNA) genes, and 8 ribosomal RNA (rRNA) genes. Sixteen genes including 5 PCGs (*ndhB*, *rpl23*, *rps12*, *rps7*, and *ycf2*), 7 tRNA genes (*trnA-UGC*, *trnI-CAU*, *trnI-GAU*, *trnL-CAA*, *trnN-GUU*, *trnR-ACG*, and *trnV-GAC*) and 4 rRNA genes (*rrn16*, *rrn23*, *rrn4.5*, and *rrn5*) are duplicated. In addition, among the 112 unique genes, 14 have one intron (*atpF*, *ndhA*, *ndhB*, *petB*, *petD*, *rpl16*, *rpoC1*, *rps16*, *trnA-UGC*, *trnG-UCC*, *trnI-GAU*, *trnK-UUU*, *trnL-UAA*, and *trnV-UAC*), and 3 have two introns (*clpP*, *rps12*, and *ycf3*).

To determine the phylogenetic position of *E. loquaiana* within Pentaphylacaceae, the whole cp genome sequences from seven species of Pentaphylacaceae and *Sladenia celastrifolia* from Sladeniaceae were downloaded from GenBank (Figure 1). The sequences were aligned with MAFFT version 7.0 (Katoh and Standley 2013). The maximum-likelihood (ML) and Bayesian inference (BI) phylogenetic trees were reconstructed using RAxML (Stamatakis 2014) and MrBayes (Ronquist et al. 2012). The ML and BI analyses generated the same tree topology (Figure 1). The result showed that the *E. loquaiana* forms a clade with *E. alate*, which is sister to the clade formed by *Euryodendron excelsum*. The complete cp genome of *E. loquaiana* reported here will provide molecular basis for the further studies on the phylogeny analysis of the genus.

**Figure 1.**
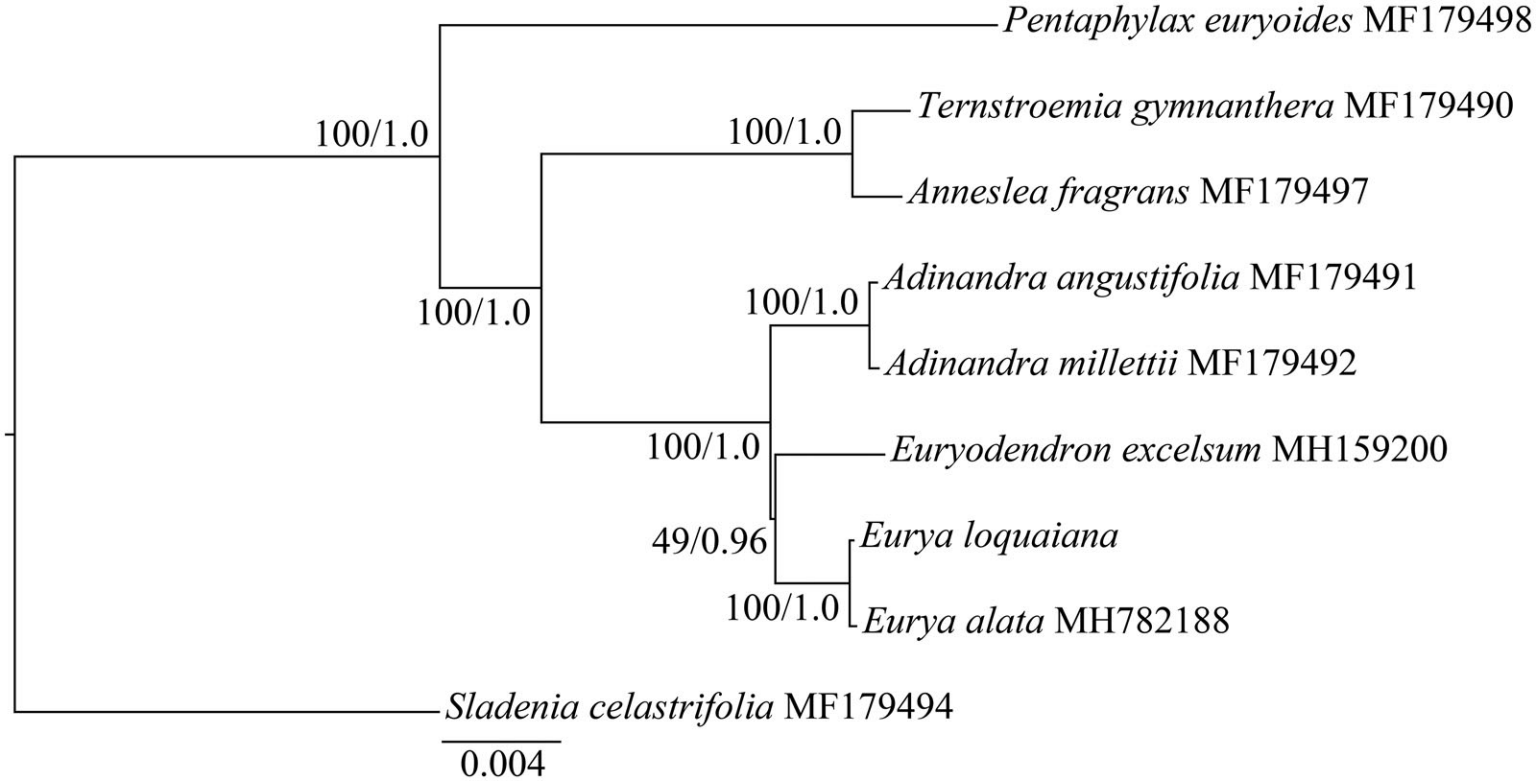
Phylogenetic tree based on nine complete chloroplast genome sequences. Numbers at nodes correspond to ML bootstrap percentages (1000 replicates) and Bayesian inference (BI) posterior probabilities. All the sequences are available in GenBank, with the accession numbers listed right to their scientific names.

## Data Availability

The data that support the findings of this study are openly available in GenBank of NCBI at https://www.ncbi.nlm.nih.gov, reference number MT038209.

## References

[CIT0001] Bankevich A, Nurk S, Antipov D, Gurevich AA, Dvorkin M, Kulikov AS, Lesin VM, Nikolenko SI, Pham S, Prjibelski AD, et al. 2012. SPAdes: a new genome assembly algorithm and its applications to single-cell sequencing. J Comput Biol. 19(5):455–477. 22506599 10.1089/cmb.2012.0021 PMC3342519

[CIT0002] Chen Y. 2010. Nectariferous plants of *Eurya* and the economic value. J Bee. 11:37.

[CIT0003] Katoh K, Standley DM. 2013. MAFFT multiple sequence alignment software version 7: improvements in performance and usability. Mol Biol Evol. 30(4):772–780. 23329690 10.1093/molbev/mst010 PMC3603318

[CIT0004] Min TL, Bartholomew B. 2007. Flora of China: Theaceae. Beijing: Science Press; St. Louis: Missouri Botanical Garden Press; vol. 12.

[CIT0005] Qu XJ, Moore MJ, Li DZ, Yi TS. 2019. PGA: a software package for rapid, accurate, and flexible batch annotation of plastomes. Plant Methods. 15:50. 31139240 10.1186/s13007-019-0435-7 PMC6528300

[CIT0006] Ronquist F, Teslenko M, van der Mark P, Ayres DL, Darling A, Höhna S, Larget B, Liu L, Suchard MA, Huelsenbeck JP. 2012. MrBayes 3.2: efficient Bayesian phylogenetic inference and model choice across a large model space. Syst Biol. 61(3):539–542. 22357727 10.1093/sysbio/sys029 PMC3329765

[CIT0007] Song JL, Yuan Y, Tan HB, Huang RM, Liu HX, Xu ZF, Qiu SX. 2017. Anti-inflammatory and antimicrobial coumarins from the stems of *Eurya chinensis*. J Asian Nat Prod Res. 19(3):222–228. 27299182 10.1080/10286020.2016.1191474

[CIT0008] Stamatakis A. 2014. RAxML version 8: a tool for phylogenetic analysis and post-analysis of large phylogenies. Bioinformatics. 30(9):1312–1313. 24451623 10.1093/bioinformatics/btu033 PMC3998144

